# Structural Characterization of Poly-l-lactic Acid (P_L_LA) and Poly(glycolic acid)(PGA) Oligomers

**DOI:** 10.3390/ijms12063857

**Published:** 2011-06-10

**Authors:** Tommaso Casalini, Filippo Rossi, Marco Santoro, Giuseppe Perale

**Affiliations:** Dipartimento di Chimica, Materiali e Ingegneria Chimica “Giulio Natta”, Politecnico di Milano, Via Mancinelli 7, 20131 Milano, Italy; E-Mails: tommaso.casalini@mail.polimi.it (T.C.); filippo.rossi@mail.polimi.it (F.R.); marco.santoro@chem.polimi.it (M.S.)

**Keywords:** DFT, torsion potential, force field, molecular dynamics, diffusion

## Abstract

Structural characterization of poly-l-lactic acid (P_L_LA) and poly(glycolic acid) (PGA) oligomers containing three units was carried out with an atomistic approach. Oligomer structures were first optimized through quantum chemical calculations, using density functional theory (DFT); rotational barriers concerning dihedral angles along the chain were then investigated. Diffusion coefficients of l-lactic acid and glycolic acid in pure water were estimated through molecular dynamic (MD) simulations. Monomer structures were obtained with quantum chemical computation in implicit water using DFT method; atomic charges were fitted with Restrained Electrostatic Potentials (RESP) formalism, starting from electrostatic potentials calculated with quantum chemistry. MD simulations were carried out in explicit water, in order to take into account solvent presence.

## 1. Introduction

Nowadays, overcoming diseases and providing worldwide medical care are high priorities. Materials science, in conjunction with biotechnology, can meet this challenge by developing safe drug delivery systems and organ implants [1].

Among the several materials suitable for biomedical applications, polyester-based polymers hold great promises because of their feasible properties and biological affinity [2], particularly those based on glycolic acid (GA) and lactic acid (both chiral isomers _L_LA and _D_LA and the raceme _L,D_LA). Primarily, they are well tolerated by the human body, as their degradation products are incorporated in the tricarboxylic acid cycle, or the Krebs’ one. Their degradation process is mainly due to hydrolysis mechanism: water diffuses into the material and breaks long chains in small oligomers which are able to diffuse within and out the polymeric matrix [3,4]. Moreover, these biopolymers possess adequate mechanical properties (*i.e.*, high Young modulus) that allow the employment of such materials in a wide range of applications. Indeed, such polymers are involved in different tissue-engineered devices, such as screws and pins for bone repair, scaffolds for tissue engineering, vascular stents or suture threads [5–11]. Currently, one of the most interesting and promising applications is the development of drug delivery systems [6,8,10,12–14]. As degradation occurs, active compounds are able to diffuse outside the polymeric matrix. These mechanisms are strongly correlated, since fast degradation kinetics implies a burst release of the active compound, while a slower hydrolysis is responsible of a more prolonged delivery of the interested molecules. A great number of experimental studies regarding the synergy between these phenomena can be found in the literature [4,10,12,15], but none of them couple experimental results with simulations at atomistic level, a test bed for a deep comprehension of involved mechanisms. Molecular dynamics could be a useful tool to study polymer structures in aqueous environment and diffusion phenomena, which can be related to both the behavior in water and in polymeric matrices. Molecular dynamics simulations can be further improved applying quantum mechanics calculations, which are helpful in determining atomic charges and force field parameters that describe the investigated system more carefully. Blomqvist *et al.* [16,17] used quantum mechanics computations, through *ab initio* and density functional theory methods, to analyze torsional barriers of aliphatic polyesters with the purpose of optimizing polymer consistent force field (PCFF) and reproducing well chain conformations. Xiang *et al.* [18] studied mechanical and thermal properties of poly(lactic acid) and poly(ethylene glycol) block copolymers by means of molecular dynamics, determining glass transition temperature (T_g_) value with COMPASS force field. Furthermore, Entrialgo-Castaño *et al.* [19] used molecular dynamics to investigate the behavior of a polymer/water interface system. They also applied quantum mechanics to analyze hydrolysis reaction, which is responsible of material degradation. Molecular dynamics is also suitable for the study of more complex systems behavior: Walter *et al.* [20] realized molecular dynamics simulations in order to determine conformation change of poly(*N*-isopropylacrilamide) hydrogels in water; Jang *et al.* [21] simulated a double network hydrogels, reproducing mechanical properties and diffusion of small molecules into the matrix; Lee *et al.* [22] studied the effect of water on mechanical properties of poly-*N*-vinyl-2-pyrrolidone-co-2-hydroxyethyl methacrylate hydrogels. Chiessi *et al.* [23,24] simulated the interactions that occur between water and a poly-vinyl alcohol hydrogel, and a thermoresponsive polymer network by means of molecular dynamics.

In this framework, the present work is focused on poly(l-lactic acid) and poly(glycolic acid), as mentioned, two extremely well known and widely studied biopolymers belonging to poly(α-hydroxy acids) family [25], often indicated only as P_L_LA and PGA. In the first part, torsional barriers through polymeric chains were obtained both through molecular mechanics and quantum chemistry computations. Molecular mechanics computations were performed with AMBER8^®^ [26] suite of programs, using GAFF (General Amber Force Field) parameters [27]. The second part deals with the computation of diffusion coefficients of monomers, which are lactic acid and glycolic acid, respectively. Indeed, the ultimate goal of this work is to test the suitability of the chosen force field when dealing with small oligomers, for what concerns both structural changes (torsional barriers) and transport phenomena (diffusion coefficients).

## 2. Results and Discussions

### 2.1. Structural Characterization

**Figure 1:**
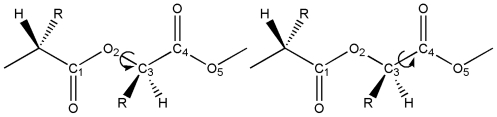
Analyzed dihedral angles; R = H for poly(glycolic acid), and R = CH_3_ for L-lactic acid.

Structural characterization was carried out by analyzing torsional potentials involving C-O and C-C rotations: the analysis was focused on computing energies with respect to the dihedral angles considered, C_1_-O_2_-C_3_-C_4_ and O_2_-C_3_-C_4_-O_5_, as also shown in Figure 1.

### 2.2. Structural Analysis of PGA

**Figure 2:**
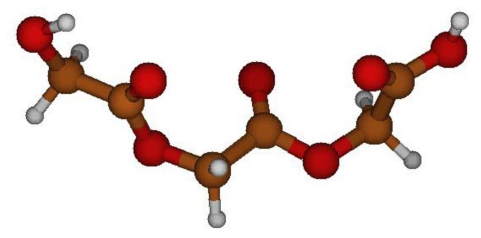
Optimized PGA structure *in vacuo* at B3LYP/6–31G(d,p) level.

PGA structure was first optimized *in vacuo* in order to obtain the global minimum energy structure, which was used for subsequent computations (Figure 2). C_1_-O_2_-C_3_-C_4_ and O_2_-C_3_-C_4_-O_5_ angle values of this minimum energy geometry were estimated equal to −87° and −164°, respectively.

Then, energy scan was done through quantum chemistry computations, changing dihedral angles with a step of 10° and optimizing the structure at every step.

**Figure 3:**
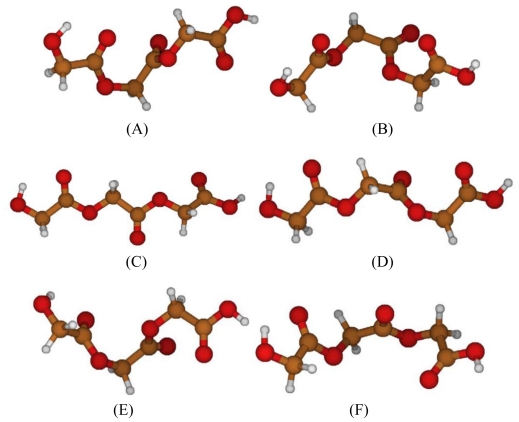
**(A)** Maximum energy PGA structure, with respect to C-O bond; **(B)** local minimum energy PGA structure, with respect to C-O bond; **(C)** global minimum energy PGA structure, with respect to C-C bond; **(D)** global maximum energy PGA structure, with respect to C-C bond; **(E)** local minimum energy PGA structure, with respect to C-C bond; **(F)** local maximum energy PGA structure, with respect to C-C bond.

Analyzing torsional potentials involving C_1_-O_2_-C_3_-C_4_ angle, energy profile shows one global minimum at −80°, which corresponds to the global optimized geometry (Figure 2), and a global maximum at 0° (Figure 3A), that offers an energy barrier of 6.87 Kcal/mol with respect to minimum. There is, moreover, a local minimum at 70° (Figure 3B), with a barrier with respect to maximum of 5.97 Kcal/mol.

The energy peak corresponds to a geometry in which the repulsion between oxygen atoms is the strongest one, since they are close and almost aligned; in the local minimum, repulsions between oxygen atoms are minimized except for the oxygen of the esters bond, which are aligned.

Referring to O_2_-C_3_-C_4_-O_5_ angle, energy profile shows a global minimum at −180° (Figure 3C) and a global maximum at −90° (Figure 3D); there are also a local minimum at −30° (Figure 3E) and a local maximum at 120° (Figure 3F).

The energy barrier between global minimum and maximum is equal to 4.27 Kcal/mol, while the barrier between the global minimum and the local maximum is equal to 4.05 Kcal/mol. The two maxima have a similar energy value; they present a difference in energy of 0.22 Kcal/mol. The energy barrier between the local minimum and the global and local maxima is equal to 2.42 Kcal/mol and to 2.20 Kcal/mol, respectively.

In the global minimum structure, oxygen repulsions are minimized, while in the global maximum, oxygen atoms are closer and in an unfavorable structure. In contrast, the local minimum structure presents a favorable conformation for oxygen atoms, except for the ones involved in esteric bonds, which are close each other. The local maximum exhibits a structure similar to the global maximum one, where oxygen interactions are not preferred.

**Figure 4:**
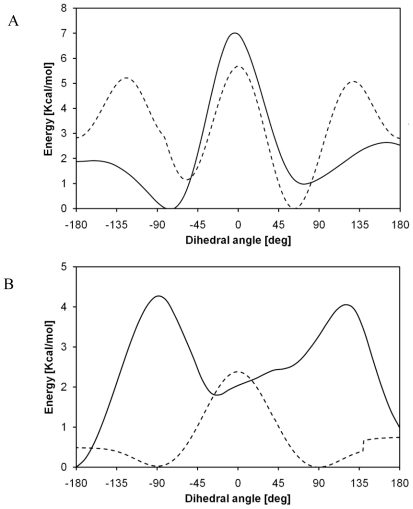
Comparison between energy barriers of C_1_-O_2_-C_3_-C_4_ angle **(A)** and O_2_-C_3_-C_4_-O_5_ angle **(B)** computed with quantum chemistry (B3LYP/6–31G (d,p), black line) and molecular mechanics (GAFF, dotted line).

The same torsional barriers were computed by means of molecular mechanics, using GAFF force field as implemented in AMBER8^®^ suite of programs. The comparison between the energy profiles computed through quantum chemistry and those obtained via molecular mechanics (Figure 4) shows a deep disagreement between the results. It can be deduced that parameterization of the dihedral term of GAFF force field cannot characterize this torsional behavior.

### 2.3. Structural Characterization of P_L_LA

P_L_LA structure was first optimized *in vacuo* in order to obtain a good guess for further computations. C_1_-O_2_-C_3_-C_4_ and O_2_-C_3_-C_4_-O_5_ angle of values of the minimum energy structure are equal to 70° and −164°, respectively.

**Figure 5:**
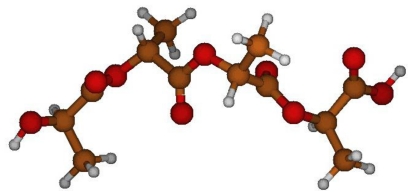
Optimized P_L_LA structure *in vacuo* at B3LYP/6–31G(d,p) level.

**Figure 6:**
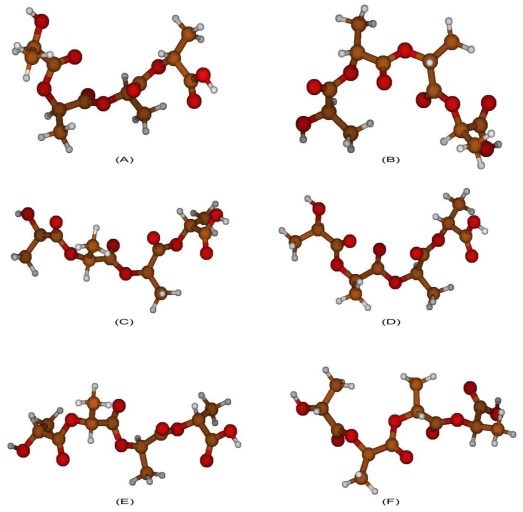
**(A)** Maximum energy P_L_LA structure, with respect to C-O bond; **(B)** maximum energy P_L_LA structure, with respect to C-C bond; **(C)** local maximum P_L_LA structure, with respect to C-O bond; **(D)** local minimum energy P_L_LA structure, with respect to C-O bond; **(E)** local maximum P_L_LA structure, with respect to C-C bond; **(F)** local minimum energy P_L_LA structure, with respect to C-C bond.

Energy profile of C_1_-O_2_-C_3_-C_4_ angle shows a global minimum at 70°, which corresponds to the optimized geometry shown in Figure 5, and a local minimum at −70°, represented in Figure 6D. There is also a global maximum at −10° (Figure 6A) and a local maximum at −120° (Figure 6C), which have a barrier of 6.96 Kcal/mol and 6.18 Kcal/mol, respectively. There is, in addition, one local maximum and one local minimum near 120°, but they were not taken into account due to the small difference in energy (less than 0.2 Kcal/mol).

The global minimum corresponds to the overall optimized geometry, where a helix conformation minimizes the interactions between oxygen atoms: in the maximum energy structure, oxygen atom positions are less favorable, increasing repulsion interactions. The same situation can be seen in the local maximum structure.

The local minimum energy geometry exhibits a helix structure similar to the overall minimum structure, but the energy is higher because of the repulsion between carbonyl oxygen atoms.

O_2_-C_3_-C_4_-O_5_ angle, energy profile shows a global minimum at −160°, which corresponds to the overall optimized geometry, and a global maximum at 110° (Figure 6B). Moreover, there are a local minimum at −40° and a local maximum at −110° (Figure 6E and 6F).

In the global maximum structure, oxygen atoms of oligomer backbone are close each other, and thus are subjected to unfavorable repulsion interaction. The global minimum energy geometry is the same as before, where the helix conformation optimizes distances between oxygen atoms.

The local minimum structure has an optimized helix structure except for two close oxygen atoms of the ester bond, while the local maximum structure does not optimize oxygen atom positions.

**Figure 7:**
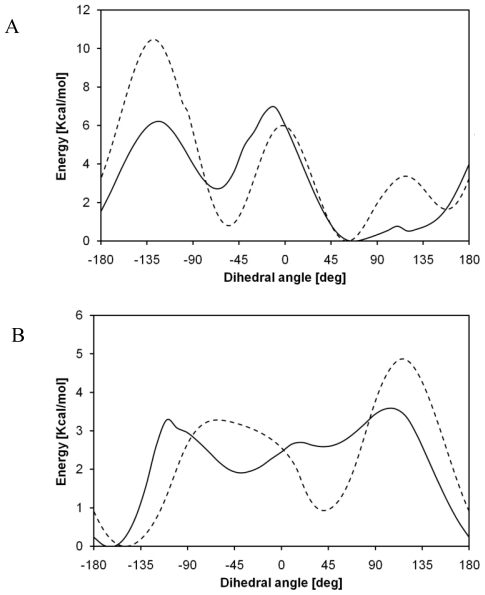
Comparison between energy barriers of C_1_-O_2_-C_3_-C_4_ angle **(A)** and O_2_-C_3_-C_4_-O_5_ angle **(B)** computed with quantum chemistry (B3LYP/6–31G(d,p), black line) and molecular mechanics (GAFF, dotted line).

**Figure 8:**
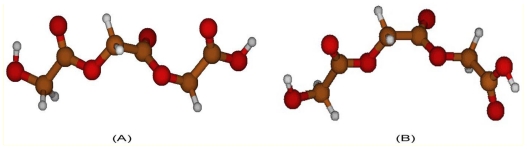
Comparison between corresponding PGA structures obtained through quantum chemistry **(A)** and molecular mechanics **(B)**.

Also, in this case, GAFF force field is not able to reproduce the torsional behavior of PLLA, as shown in Figure 7. As regards C_1_-O_2_-C_3_-C_4_ angle, the global minimum is well characterized, but the other configurations are not in agreement with quantum chemistry computations. Energy profile concerning O_2_-C_3_-C_4_-O_5_ angle shows a bad agreement with quantum chemistry torsional barriers. In order to understand the poor agreement of energy profiles, structures obtained through molecular mechanics can be compared with the corresponding ones characterized by means of quantum chemistry. It can be seen, indeed, that molecular mechanics geometries are very different from quantum chemistry ones. Even if a great number of minimization steps are performed, in order to obtain minimum energy structures using atoms coordinates as variables, the system is not able to relax properly. This can be related to a parameterization that is not suitable for such systems; GAFF force field, indeed, is intended to be used only with small organic molecules, and not with polymers. For the sake of completeness, an example referring to the PGA global maximum with respect to O_2_-C_3_-C_4_-O_5_ angle is here reported (Figure 8): a deep disagreement is evident since this structure represents a global minimum for molecular mechanics, while it is a global maximum for quantum chemistry.

### 2.4. Diffusion Coefficients Computation

Diffusion coefficients were computed by means of Einstein equation [28], starting from molecule trajectories:

(1) $$D={\text{lim}}_{t\to \infty }\frac{1}{6t}\langle {\left(r(0)-r(t)\right)}^{2}\rangle$$

**Figure 9:**
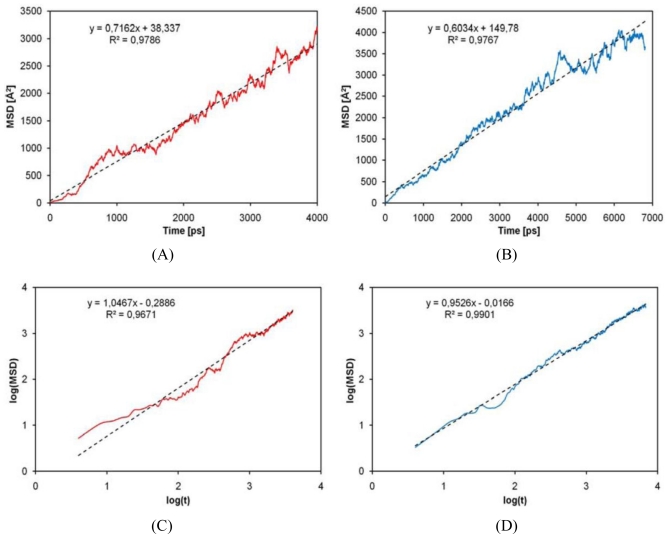
(**A**) *t vs.* mean square displacement (MSD) plot for glycolic acid; (**B**) *t vs.* MSD plot for lactic acid; (**C**) log(t) *vs.* log(MSD) plot for glycolic acid; (**D**) log(t) *vs.* log(MSD) plot for lactic acid.

Where *D* is the diffusion coefficient, *t* is the time, and *r*(*t*) is the position of the molecules at time *t*. The term between brackets in equation (1) is also known as the mean square displacement (MSD), that is, the quadratic distance covered by the molecule with respect to its initial position, *i.e.*, *r(0)*. In particular, the motion of the center of mass of the molecules was here considered, while angle brackets indicate that mean square displacement used in the computation of diffusion coefficient is averaged on all solute molecules. The limit operator has a physical meaning: the simulation must be long enough in order to attain Brownian motion regime, since the equation is valid only under this hypothesis. Basically, mean square displacement plotted against time gives a straight line with a slope equal to 6D. In order to verify the Brownian motion regime, and thus the validity of its linear relationship, it is possible to plot log(MSD) against log(*t*): if the slope is equal to one, then MSD is a linear function of time and Einstein equation is valid [28]. Figure 9B shows log(*t*) *vs.* log(MSD) estimated for the lactic acid monomer. Computed diffusion coefficient for lactic acid is equal to (1.006 ± 0.032) × 10^−5^ cm^2^/s, and it is in good agreement with the experimental value of 0.993 × 10^−5^ cm^2^/s reported by Ribeiro *et al*. [29]. The slope of the log(*t*) *vs.* log(MSD), as shown in Figure 9D, is close to one and the value obtained is surely representative of the behavior of the system. Figure 9A shows *t vs*. MSD plot for glycolic acid, whose computed self-diffusion coefficient is equal to (1.194 ± 0.062) × 10^−5^ cm^2^/s.

Up to our best knowledge, an experimental self diffusion coefficient for glycolic acid is not available in literature, but the computed result can be considered reasonable, since it is very similar to the one of lactic acid. The slope of log(*t*) *vs.* log(MSD) is very close to one and thus diffusion regime is reached.

## 3. Simulation Details

### 3.1. Structural Characterization

Starting structures were P_L_LA and PGA oligomers composed by three monomeric units. First, structure geometries were optimized *in vacuo* by means of density functional theory (DFT) using a Becke 3 parameters [30] and Lee-Yang-Parr [31] (B3LYP) functional for exchange and correlation energy, and a 6–31G(d,p) basis set [32], implemented in Gaussian03 suite of programs [33]. In this way, it was possible to find an overall optimized structure, which represents an energy minimum used as an initial estimate. Dihedral angles were changed stepwise from −180° to 180° at 10° intervals. Structure optimization was carried out at each step. Indeed, while the dihedral angle of interest was restrained to a certain value, the molecule geometry was optimized in order to obtain the minimum energy structure related to that dihedral angle value. In this way, it was possible to obtain the most probable structure at every step. In order to investigate torsional barriers by means of molecular mechanics, GAFF force field [27] implemented in AMBER8^®^ suite of programs [26] was chosen. Optimized P_L_LA and PGA structures were placed *in vacuo*, changing dihedral angles values from −180° to 180° with a step of 5°. At every step, a minimization procedure of 10^6^ cycles was carried out, applying a restrain to the investigated dihedral angles. As in the DFT approach, while the dihedral angle of interest was restrained, the molecule geometry was optimized through energy minimization in order to obtain the most probable structure with respect to that dihedral angle value. In particular, 100,000 minimization cycles were run with the steepest descent algorithm, while the remaining ones were accomplished through conjugate gradient algorithm, which is more efficient when the system is close to an energy minimum. Atomic charges were computed starting from electrostatic potentials (ESP) calculated through quantum chemistry at B3LYP/6–31G(d,p) level; then, charges were fitted following RESP (Restrained Electrostatic Potentials) [34] formalism. As mentioned, this analysis aims to assess whether GAFF force field is able to reproduce torsional barriers, and thus if it can provide reliable predictions about oligomers conformations. Analysis was carried out *in vacuo* in order to neglect the solvent effect [35].

### 3.2. Molecular Dynamics Simulations

In order to provide further proof as to the suitability of the adopted method, *i.e.* the chosen force field, molecular dynamics simulations were used to compute monomers’ self diffusion coefficients. Lactic acid and glycolic acid structures were first optimized by DFT calculations in implicit water. Becke 3 parameters [30] and Lee-Yang-Parr [31] (B3LYP) functions for exchange and correlation energy, and 6–31G(d,p) basis set [32] were adopted for the geometry optimization, as implemented in Gaussian03 suite of programs [33]. Water was modeled with the integral equation formalism polarizable continuum model [36] (IEFPCM) at a temperature of 300 K. In order to compute atomic charges, electrostatic potentials (ESPs) were calculated at the B3LYP/6–31G(d,p) level; charges were then fitted through RESP [34] formalism. Ten molecules of each compound were solvated in a cubic box with a lateral size of 60 Å, containing about 9000 explicit TIP4P water molecules [37]; cutoff for non-bonded interactions was set to 15 Å, and simulations were carried out using periodic boundary conditions. Long-range electrostatic interactions were evaluated through the Particle Mesh Ewald method. As mentioned, simulations were performed using GAFF force field as implemented in AMBER8^®^ suite of programs: in particular, the following simulation protocol was adopted. First of all, a 2000-cycle minimization, in which the solute is restrained with harmonic potential k(Δx)^2^ (where Δx is the displacement and k is the force constant, equal to 500 Kcal mol^−1^ Å^−2^), was performed in order to cut out bad contacts which derive by the random placing of solvent molecules. 1000 minimization steps were realized through the steepest descent algorithm, while the remaining 1000 were accomplished through the conjugate gradient algorithm, which is more efficient when the system is close to an energy minimum. Then a 3500-cycle minimization was performed in order to minimize the energy of the whole system, without restraints. In this case, 2000 minimization steps were done through the steepest descent algorithm, and the remaining ones were realized with conjugate gradient algorithm. The temperature was raised from 0 K to 300 K by a simulated annealing of 20 ps at constant volume, imposing a weak restraint (k = 10 Kcal mol^−1^ Å^−2^) on the solute with the purpose of avoiding wild fluctuations, and afterwards the system was relaxed with a 100 ps run at constant pressure in order to reach the correct density of the solution. Finally, molecular dynamics simulations were carried out, investigating a time span of 5 ns. SHAKE algorithm was used for all covalent bonds involving hydrogen atoms, thus allowing a time step of 2 fs. Simulations were run under steady conditions (300 K and 1 atm). Temperature and pressure were controlled using Langevin dynamics (with a collision frequency equal to 1.0 ps^−1^) and isotropic position scaling, respectively.

## 4. Conclusions

Torsional barriers involving C-O and C-C bonds were investigated for PGA and PLLA oligomers, by means of quantum chemistry and molecular mechanics. The analysis shows that oligomers were allowed to conform in different structures, since there are also local minima in the energy profiles. Comparing torsional barriers computed through quantum chemistry with the ones obtained by means of molecular mechanics, it could be stated that the GAFF force field chosen is not able to describe the structural behavior. Such disagreement with quantum chemistry profile is related to parameters of dihedral term of the force field, which are not suitable for such analysis. Indeed, even if a large number of minimization steps were performed through molecular mechanics, structures were not able to relax properly. However, GAFF force field is surely suitable to characterize transport phenomena in water environment, since the computed diffusion coefficient for lactic acid satisfactorily matches the experimental one. This confirms the suitability of the method used to compute atomic charges, and that long range interactions are determined in a proper way.
